# Solar Power Can Substantially Prolong Maximum Achievable Airtime of Quadcopter Drones

**DOI:** 10.1002/advs.202001497

**Published:** 2020-08-19

**Authors:** Ching‐Fuh Lin, Ta‐Jung Lin, Wei‐Sheng Liao, Hsiang Lan, Jiun‐Yu Lin, Chi‐Han Chiu, Aaron Danner

**Affiliations:** ^1^ Graduate Institute of Photonics and Optoelectronics Graduate Institute of Electronics Engineering Department of Electrical Engineering Innovative Photonics Advanced Research Center National Taiwan University 1, Sec. 4, Roosevelt Road Taipei 10617 Taiwan; ^2^ Taipei Municipal Jianguo High School No. 56, Nanhai Road Taipei 10066 Taiwan; ^3^ Department of Electrical Engineering National Taiwan University 1, Sec. 4, Roosevelt Road Taipei 10617 Taiwan; ^4^ Department of Electrical and Computer Engineering National University of Singapore University Town Stephen Riady Centre, 2 College Avenue West, #01‐03 Singapore 138607 Singapore

**Keywords:** airtime, power‐to‐weight ratio, quadcopters, solar cells, sunlight

## Abstract

Sunlight energy is potentially excellent for small drones, which can often operate during daylight hours and fly high enough to avoid cloud blockade. However, the best solar cells provide limited power, compared to conventional power sources, making their use for aerial vehicles difficult to realize, especially in rotorcraft where significant lift ordinarily generated by a wing is already sacrificed for the ability to hover. In recent years, advances in materials (use of carbon‐fiber components, improvement in specific solar cells and motors) have finally brought solar rotorcraft within reach. Here, the application is explored through a concise mathematical model of solar rotorcraft based on the limits of solar power generation and motor power consumption. Multiple solar quadcopters based on this model with majority solar power are described. One of them has achieved an outdoor airtime over 3 hours, 48 times longer than it can last on just battery alone with the solar cells carried as dead weight and representing a significant prolongation of drone operation. Solar‐power fluctuations during long flight and their interaction with power requirements are experimentally characterized. The general conclusion is that solar cells have reached high enough efficiencies and can outperform batteries under the right conditions for quadcopters.

## Graphical abstract

A quadcopter using on‐board solar module to harvest sunlight outdoors achieves over 3 h airtime, the longest for quadcopters without using chemical fuels and about 48 times more than using battery only. The accumulated charge generated from sunlight is 33 300 mAh, which would require a battery much heavier than the solar module and beyond the thrust capability to carry.

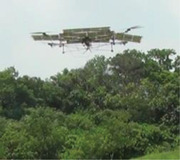

## 1 Introduction

Transportation accounts for about 25% of global energy consumption,^[^ ^1^ ^]^ and replacing fossil fuels in transportation with renewable energy sources is paramount in reducing humanity's carbon footprint.^[^ ^2^ ^]^ Although zero‐emission electrical vehicles have recently attracted attention, their energy still largely originates from fossil fuels. Transportation represents a particular challenge, because vehicles must carry their own power sources, and gasoline and other liquid fuels^[^ ^3^ ^]^ have high energy densities which are difficult to match. In the case of rotorcraft where there is no wing to continuously generate lift, any additional weight carried on board must be compensated by additional continuous thrust simply to hover in place.

Solar energy is appealing in transportation, because the power source (the sun) does not need to be carried around; only the power conversion system (solar panel) is needed. This potential use of solar energy is particularly interesting in aircraft, which usually have a clear view of the sky. How does the mass of a solar cell and the associated energy it can supply compare with the energy densities of fuels or batteries? Since the comparatively low power associated with solar cells is not commonly appreciated, one of the greatest aviation achievements of the 20th century was the demonstration of solar‐powered flight in a heavier‐than‐air fixed‐wing vehicle.^[^ ^4^ ^]^ As the energy density of gasoline is 47 MJ kg^−1[^ ^5^ ^]^ and that of a lithium ion only 0.7–0.9 MJ kg^−1^,^[^ ^6^ ^]^ there is a nearly 50‐fold difference. (This difference is partially bridged to an overall factor of ≈10 in electric cars because motors have a much higher mechanical conversion efficiency compared to gasoline engines.^[^ ^7^ ^]^) But certain Si solar cells on the market today have a specific power > 572 W kg^−1^,^[^ ^8^ ^]^ or 2.1 MJ kg^−1^ if used optimally for an hour in full sunlight. As it is slightly better than the energy density of batteries, it is clear why solar energy is promising in aviation; difficulties arise because solar cells must be spread over a large surface area, requiring additional structure to support and protect them, and because they will never operate at this peak laboratory efficiency when flown under variable conditions outdoors.

Despite these difficulties, solar powered flight in rotorcraft has been demonstrated, albeit with very short (<2 min) flight times.^[^ ^9^, ^10^, ^11^, ^12^ ^]^ Fixed‐wing solar aircraft have achieved perpetual flight;^[^ ^13^, ^14^, ^15^, ^16^, ^17^, ^18^ ^]^ the use of solar power in such airplanes is relatively straightforward because a wing's surface is a natural position for solar panels, but fixed‐wing aircraft are not easy to operate and need well‐trained pilots and dedicated runways. Instead, rotorcraft that can take off and land vertically are much easier to operate, but the required power is significantly greater.^[^ ^19^, ^20^ ^]^ Moreover, the design of conventional rotorcraft such as quadcopters leaves little obvious room for solar cells, since propellers sweep out large surface areas which must not be blocked. In one previous quadcopter which achieved stable 100% solar flight,^[^ ^10^ ^]^ the large surface area required by its solar cells made the aircraft sensitive to wind and flight control was difficult which limited flight time. Here, we experimentally achieve much longer airtimes in quadcopters, following quantitative modeling of power consumption and generation of solar power on rotorcraft. We discover that the aerodynamics of a quadcopter with a large on‐board solar module are not conducive to long duration flight, because its presence significantly worsens power fluctuations; meeting peak power demands over a long duration flight is more challenging than in short flights in good conditions.

With those challenges overcome through quantitative evaluation of quadcopter power consumption and generation from solar modules based on a model described in Section 2.1, as well as stabilization of the operating voltage of the solar module as described in Section 2.4, an airtime of over 2 h has been achieved in several solar‐powered quadcopters in our experiments, with the longest being 3 h and 28 min. Although the quadcopter in particular had a small battery onboard in addition to the solar cells, this time is 48.7 times longer than what could have been achieved on battery alone (with the solar panel present but disconnected). The total solar energy provided over that time by the 1.02 kg solar module was 33 300 mAh, which if instead had been provided by an onboard battery would have required a battery heavier than 3 kg (insufficient thrust to carry).

## 2 Results

### 2.1 Concise Model for Power Analysis

Rotorcraft such as helicopters^[^ ^21^, ^22^ ^]^ and quadcopters^[^ ^23^, ^24^, ^25^ ^]^ have been improving for many years. The working principles of quadcopters in particular have been extensively described in literature,^[^ ^26^, ^27^, ^28^, ^29^, ^30^, ^31^, ^32^, ^33^ ^]^ with most attention related to balance control and stability.^[^ ^26^, ^27^, ^28^, ^29^ ^]^ A few detailed analyses exist which relate to calculation of thrust, but with different effective efficiency.^[^ ^31^, ^32^ ^]^ In the unique case of solar power, we must consider factors which affect power generation and consumption in a rigorous way.

We begin with two important equations; a detailed derivation is given in the Supporting Information. The first equation relates the electrical power *P*_t_ delivered to the motors to the lifting force (the upward thrust) *F*_t_

(1) $$\begin{array}{c} P_{t}=\frac{1}{2\eta }\frac{1}{\sqrt{A_{t}\rho }}{F_{t}}^{3/2} \end{array}$$

where *A*_t_ is the total surface area swept by the propellers, *ρ* is the density of air, and* η* is the efficiency of converting input electrical power to downward air‐flow power. The second equation relates the power which must be supplied by the solar cells *P*_s_ and the total weight of the solar quadcopter *W* during static hover

(2) $$\begin{array}{c} P_{s}=r\left(W-W_{f}\right) \end{array}$$

where *W*_f_ is the fuselage weight (quadcopter weight without solar module) and *r* is the power‐to‐weight ratio (PWR) of the solar module, the most critical parameter in determining the possibility of using sunlight as the main power source in any particular rotorcraft. The quadcopter will achieve static hover when *F*_t_ equals the total weight *W*.

**Figure 1:**
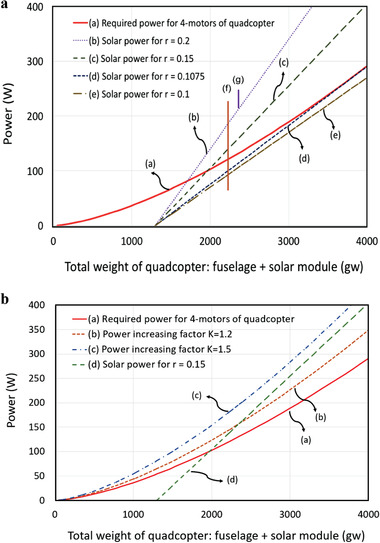
Curve (a) shows necessary thrust, curves (b–e) show hypothetical power available from solar modules with different PWR, line (f) shows experimental power variation for a 2230 g solar‐quadcopter operated under solar power only, with huge fluctuation, and line (g) shows the power range of a 2350 g solar quadcopter operating under varying sun orientation on September 19, 2018 at a latitude of 24.5^°^. For curves (b–e), only (b) and (c) intersect (a), indicating sufficient power. (b) Required thrust is varied in (a–c) with an example PWR in (d). Only (a) and (b) intersect (d), indicating sufficient power.

The minimum required quadcopter power and the power generated by the solar module as a function of total weight are plotted together in **Figure** **1**a in a hypothetical example aircraft. Curve (a) is the quadcopter's required power (*P*_t_) with an example case of four MAD5005 motors and 18 in. propellers, calculated according to Equation (1). Curves (b) and (c) show output power generated from two hypothetical solar modules (*P*_s_) with different PWR (*r*) for an assumed propeller diameter of 18 in., an efficiency *η* of 47%, and a fuselage weight of 1300 g. In Figure 1a, the abscissa is the total weight of the quadcopter, including the fuselage weight of 1300 g and the weight of the solar module. As observed in those curves, a larger *r* results in greater power generated by the solar module. For curve (e) with *r* = 0.1 W g^−1^, the available solar power *P*_s_ is always less than the required power *P*_t_, so the aircraft cannot lift its own weight. Curves (b) and (c) correspond to a solar‐generated power *P*_s_ that is greater than the required power *P*_t_ but only when the solar module is large enough.

The minimum *r* required is at the condition when the line *P*_s_ is tangent to the curve of *P*_t_, corresponding to *r*_min_ ≈ 0.1075 W g^−1^ in the example of Figure 1a as shown in curve (d). In practice, because a quadcopter will continuously shift its pitch somewhat flight, its solar cells will often receive less light than expected for “static” hover. Thus *r* must be somewhat larger than *r*_min_ to ensure that the solar power is actually sufficient most of the time. Curves (b) and (c) then illustrate a situation where a quadcopter's power needs can be completely met by solar cells, when *P*_s_ > *P*_t_. In the range shown in Figure 1a, the excess power (*P*_s_–*P*_t_) increases with the weight and the size of the solar module, so the larger the solar module, the easier it should be for such a module to supply the necessary power provided that *r* > *r*_min_ is satisfied. This excess power increase will also provide a quadcopter some payload capability. On the other hand, because the power consumption increases super‐linearly, while the power generation increases linearly, only a certain range of weight works, but the range could be pretty large. As a matter of fact, there should be a maximum value of the excess power (*P*_s_ − *P*_t_). Because this maximum value occurs at quite a heavy weight, much larger than what we experimented with, we will not elaborate more on this situation here. The *r* values considered in curves (b) and (c) are less than 0.429 W g^−1^, which corresponds to bare c‐Si solar cells without lamination or structural support; in curve (b) for example with *r* = 0.2 W g^−1^, an additional 2.66 g of structural support can be included for every watt supplied. This is plausible, but would still results in a flimsy cell array compared to commercial solar modules which are extremely sturdy by comparison.^[^ ^33^ ^]^ This relationship between aircraft weight and flyability has been borne out experimentally in a series of quadcopters of various sizes and weights by one of the authors (12, 16, 48, 64, 96, 124, and 148 cells, with respective aircraft weights of 401, 600, 1614, 2149, 2298, 2568, and 2604 g).^[^ ^11^ ^]^  Without an onboard battery, only the largest aircraft of 148 cells could lift its own weight in stable flight. In that aircraft, 360 W was delivered by a 1225 g solar module (cells, busbars, and fasteners), giving a realistic *r* = 0.29 W g^−1^, versus a limiting maximum *r* = 0.55 W g^−1^ (bare cells) and a limiting minimum *r* = 0.14 W g^−1^ (entire aircraft counted as “solar module” and *W*_f_ = 0).

The preceding analysis assumes only static power, 4 motors, in hover. In practice, a moving quadcopter's control system continually makes adjustments to its attitude to maintain position. Outdoor conditions (wind gusts and pressure changes) cause dynamic instabilities which increase actual power needed beyond the simple model above. The actual power consumption, *P*_ac_, can be written as *P*_ac_ = *KP*_t_, where *K* is a power increase factor to account for this (*K* ≥ 1) and *P*_t_ is the static power given in Equation (3). This increases the solar module's minimum required PWR. The situations for three different *K* are illustrated in Figure 1b. Curve (a) is the baseline condition at *K* = 1. As this curve intersects curve (d), the quadcopter has adequate thrust to lift its own weight. In curve (b) where *K* = 1.2, this is still possible. However in curve (c), as *K* = 1.5, a solar module with PWR of *r* = 0.15 W g^−1^ produces insufficient power, since curves (c) and (d) never intersect. *K* is influenced by weather expectations and the quadcopter's configurational stability.

### 2.2 Implementation of Solar Cells on Quadcopters

By considering the use of candidate motors and propellers with experimentally measured thrust data, along with candidate solar cells of known efficiency, the above procedure helps determine the necessary minimum solar array size and aircraft weight. There are many types of solar cells.^[^ ^34^, ^35^, ^36^, ^37^, ^38^ ^]^ State‐of‐the‐art multijunction solar cells have reached efficiencies beyond 40%,^[^ ^36^, ^37^, ^38^ ^]^ but are very expensive, making single‐junction solar cells^[^ ^38^, ^39^, ^40^, ^41^ ^]^ more attractive. Such GaAs crystalline and thin‐film solar cells have both reached efficiencies over 29%,^[^ ^38^, ^40^ ^]^ but they are also costly. In comparison to other types, c‐Si solar cells are more affordable and have higher efficiency^[^ ^39^ ^]^ than other thin‐film solar cells,^[^ ^38^, ^41^ ^]^ reducing the surface area required for a given output power. SunPower Maxeon 5 in c‐Si solar cells were chosen in all our aircraft because both *p*‐side and *n*‐side contacts are on the backsides of the cells and can be tabbed from the side, reducing busbar weight.

**Figure 2:**
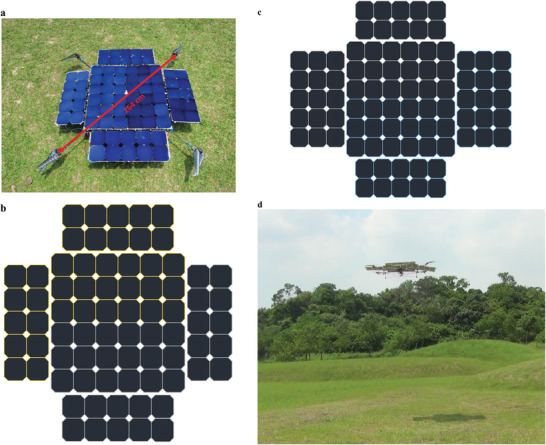
a) Photo of the solar quadcopter with 5S solar modules. b) Layout of the 5S solar panel, giving a voltage between 18.5 and 21 V. c) Layout of the 6S solar panel, giving a voltage between 22.2 and 25.2 V. d) Photo of the 6S quadcopter flying.

We have made several solar quadcopters and here we report two of them. The first one has 76 solar cells, split into two modules to match a 5S motor voltage, where 1S voltage means 4.2V, defined for typical batteries used to power drones (with a 5S voltage is 5 times 4.2 V and a similar meaning for 6S). The second one has 86 solar cells, also separated to two modules to match the 6S voltage. The motors are MAD5005, which can be operated with 5S and 6S voltages.^[^ ^42^ ^]^ The solar quadcopters, solar‐cell layouts, and a flight photo are shown in **Figure** **2**a–d. Their composition is given in **Table** **1**. Fabrication details are given in the Experimental Section.

**Table 1:** Composition of two example solar quadcopters

Solar quadcopter	Voltage	Fuselage weight [g]	Solar‐module weight [g]	PWR of solar module [W g^−1^]	Number of solar cells	Motor	Propeller
I	5S (18.5–21.0 V)	1330	900	0.268	76	MAD5005	2478
II	6S (22.2–25.2 V)	1330	1024	0.268	86	MAD5005	2478

### 2.3 Fluctuation of Solar Power and Influence During Flight

**Figure 3:**
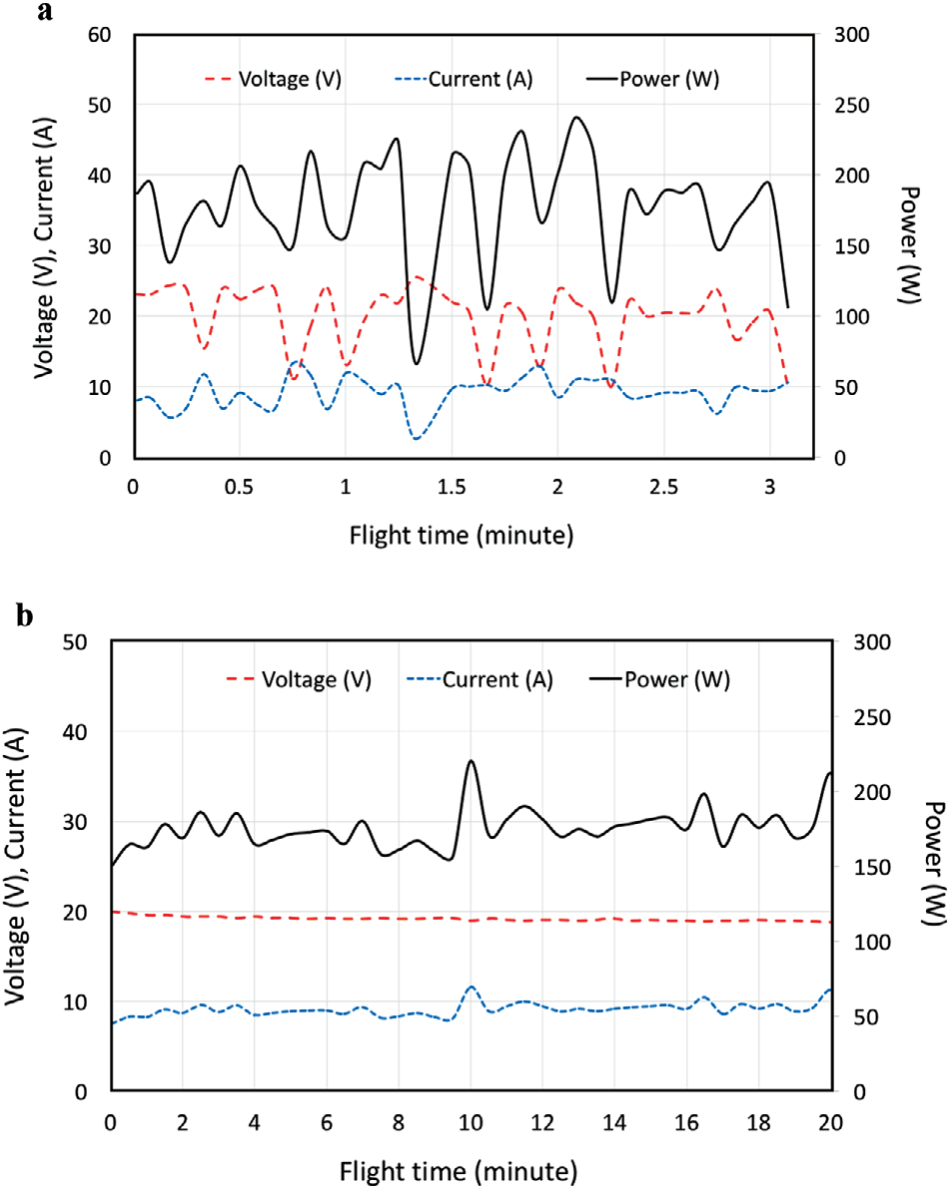
Voltage, current, and power during flight: a) with solar power only; b) with solar array and a battery connected in parallel.

Both solar quadcopters were operated outdoors in natural sunlight on multiple occasions, with a data link installed to monitor and record real‐time voltage and current using a smart phone. The copter was set to autopilot with altitude hold and position hold. **Figure** **3**a shows one set of recorded data in the 6S aircraft for a flight time of 3 min and 10 s. The voltage varies between 10 and 23.8 V with an average of 19.9 V. The variation of the current is also huge, from 2.6 to 11.8 A with an average of 9.26 A. The variation is greater than 50% of the average. In addition, voltage and current curves reveal that when the voltage increases, the current decreases. This is because of the inverse relationship between voltage and current in a solar cell, particularly when it is operated near its maximum power point. The corresponding power variation is huge, with a minimum of 66 W and a maximum of 231 W. The average is 177 W and the variation is greater than 50% of the average power. Such a power range is also plotted in Figure 1a, line (g), for easy comparison with the theoretical curves. As shown in line (g), this power range corresponds to a large variation of the PWR with some portion even less than the *r*_min_ of 0.1075 W g^−1^. The variations are troublesome because they may lead to situations where peak power demands cannot be met, resulting in flight instability or electronics brownouts. Figure 3a also shows that the durations of the peaks are mostly over 5 s with a couple of enormous surges lasting tens of seconds, indicating that a short flight^[^ ^9^ ^]^ cannot clearly reveal the extent of expected fluctuations.

These fluctuations have also been observed in a different 48‐cell quadcopter in Singapore making use of identical Sunpower cells.^[^ ^11^ ^]^ In a 1 min hover‐within‐ground‐effect with solar power alone and no battery, current supplied varied between 0.4 and 11.1 A around an average of 4.2 A.

### 2.4 Methods to Avoid Power Fluctuation

To stabilize the output of the solar modules, we had considered and tested a maximum power point tracker (MPPT),^[^ ^43^ ^]^ but an MPPT which could handle the power levels present for all our aircraft would be too heavy. Moreover, in situations where there would be excess power available from the solar cells than that needed by the motors, an MPPT cannot function without having an energy storage device to which the excess power can flow. We instead installed a small‐capacity battery, so the voltage of the solar module would be biased reasonably close to its maximum power point by the battery. Figure 3b shows that in this configuration the voltage of the 6S aircraft remains between 19 and 20 V, with less than 5% variation. The current varies between 7.5 and 11.6 A with maximum variation reduced to 4.1 from 9.2 A. The power now varies between 150 and 220.4 W with maximum variation reduced from 165 to 70.4 W. The fluctuations are thus significantly improved from the solar‐only Figure 3a case. The improvement was also obtained for the 48‐cell quadcopter in Singapore when a battery was added and hover‐out‐of‐ground‐effect was achieved, current supplied by solar cells varied only between 9.3 and 10.8 A around an average of 10.3 A. A small battery can thus substantially stabilize power flow.

Because the quadcopter must dynamically adjust its attitude, the current and the power (not voltage) still fluctuate. This cannot be avoided. Figure 3b shows that the average consumed power is around 170 W, which is greater than the statically evaluated 122 W for 4 motors. It indicates that stabilizing the quadcopter during actual flight to maintain balance increases motor power requirements by about 40%, corresponding to a power increase factor *K* = 1.4.

For the average power of 170 W with stabilization using a battery, the solar module is 5S2P. Its weight is 900 g, corresponding to *r* = 0.189 W g^−1^, obtained from the average power consumption. The maximum power from the solar cells is only 231 W, so the *r* of the solar module could be about 0.257 W g^−1^. If the flight had been carried out under more direct sunlight instead of in September at a latitude of 24.5^°^, *r* could be further increased. Certainly, the output power of the solar module varies with time, date, and location as well as weather conditions, so *r* changes. However, the values of the maximum *r* and the average *r* are still meaningful for the flight evaluation of the solar quadcopter.

Compared to the fully solar‐powered quadcopters developed previously,^[^ ^10^ ^]^ the current design targets long time durations. The Singapore design of ref. [10] had a targeted goal of having solar power sufficient to power the quadcopter without batteries on board at all, so the design had to cater for peak power demand; the resulting design was less stable, larger, and could not be folded and transported easily to where the forecast sunshine was good for hours. The maximum flight time demonstrated was under 2 min. In fact, when that aircraft^[^ ^10^ ^]^ was constructed, the Singapore group members did not realize the large extent that catering for peak power demands influenced the design, and adopted an experimental “increase the surface area until it works without a battery” approach.^[^ ^11^ ^]^ It did not include telemetry, so the time‐dependent power demands were never studied. The current work shows that a modification of the power supply with even a small onboard battery greatly improves the performance, achieving much longer stable flight, with less surface area, allowing more solid construction.

### 2.5 Hours of Flight under Sunlight

Several of our solar quadcopters have achieved long airtimes. Two examples corresponding to those in Table 1 are elaborated here. The first, with the 5S aircraft (76 solar cells), has a 5S2P 18 650 battery of 6.4 Ah charge capacity. With the solar cells disconnected but still present, 19 min of airtime could be achieved with battery power alone. With the solar module connected in parallel with the battery, this was extended to 145 min, an improvement by a factor of 7.6. The consumed charge was 25 600 with 6400 mAh provided from the battery, so the solar module provided 19 200 mAh. The solar module weighs 900 g. If this weight were replaced with a battery of similar mass, then it would give 10 800 mAh (5S voltage). Plus the 6400 mAh, the total charge would be 17 200 mAh. We expect the flight time would be around 97 min, still less than what solar energy could provide.

**Figure 4:**
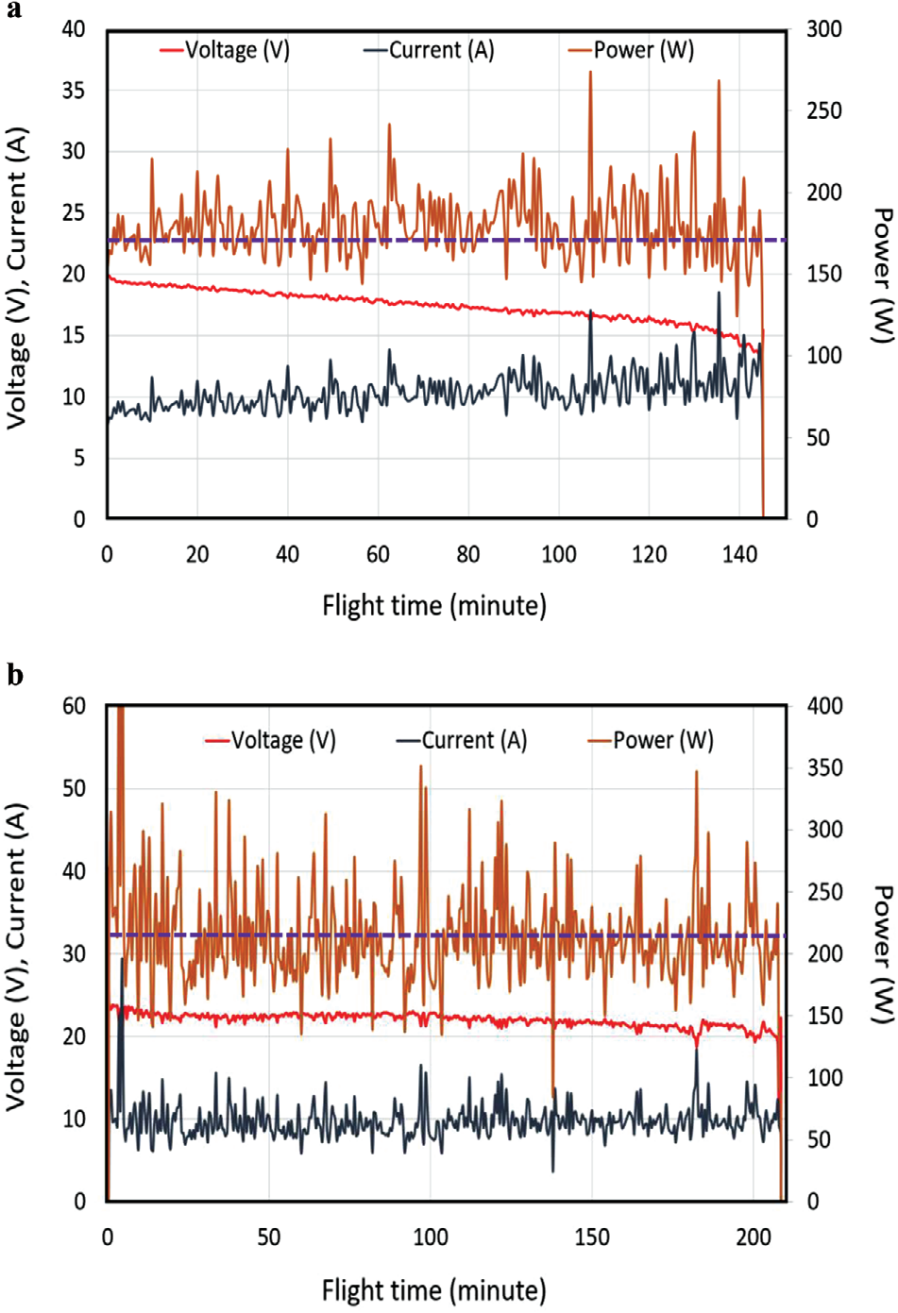
The variation of voltage, current, and power with time: a) in the 76‐cell 5S quadcopter; b) in the 86‐cell and 6S quadcopter. (The purple dashed lines indicate the mean power: 179 W for (a) and 215 W for (b).

The second example pertains to the 6S aircraft (86 solar cells), which used a very small 6S1P 18 350 battery (881 mAh). With the solar cells disconnected but still present, only 4 min 17 s of airtime could be achieved with battery power alone. With the solar module connected, the airtime was extended to 208 min, an improvement by a factor of 48.7. The voltage and the current in both examples were recorded and are shown in **Figure** **4**a,b for the 5S and 6S aircraft, respectively. For the 6S case, the total energy delivered by the solar module over the 208 min was 33 300 mAh. If a 6S battery had hypothetically been needed at this rating, its weight would be over 3 kg, much more than that of the 1.02 kg solar module. On the other hand, if a battery of 1.02 kg were used instead, it would have a 10 200 mAh charge capacity and might give about 1 h flight time, less than one third of that using solar energy. This indicates the advantage of using sunlight for quadcopters flying outdoors.

In each of the preceding two examples, the quadcopters were programmed to autonomously maintain altitude and position without human intervention. In the 5S quadcopter, during the 145 min recorded, the solar power was insufficient to fully power the aircraft, and the battery's charge was slowly consumed, as shown by a steadily dropping voltage in Figure 4a. By comparison, the 6S aircraft had sufficient solar power but only around noon. At the beginning, near 10:30 am, the position of the sun led to there being insufficient solar power production and the battery charge was consumed, leading to a slight drop of the voltage. When the time approached noon, the solar power increased and became more than the consumption. As a result, the battery was recharged, reflected by an increase of the voltage, as shown between about 35–100 min in Figure 4b. At the 100 min point (12:10 pm), there should nominally have still been enough power, but a thin cloud changed the situation and the battery again was discharged, albeit slowly, until 2:00 pm. The resulting 208 min airtime is, to the best of our knowledge, the longest reported for quadcopters without using chemical fuels. A protection circuit prevented the 6S1P 18 350 battery from being damaged during recharge. A 64X speed video of the 6S example is available (https://youtu.be/2wqc0XeeeQ0, Unlisted). The altitude‐hold functionality of the quadcopter makes use of a barometric pressure measurement, so a variation over ≈3 m of altitude can be seen in the video due to dynamic pressure changes being interpreted by the control system as altitude changes. When the quadcopter sometimes gets within a half meter or so of the ground, it is possible that the motors’ power needs are a bit reduced due to the ground effect, but we cannot easily quantify the extent that this may have lengthened the airtime achieved. From the video, the aircraft spent > 85% of the time higher than a half meter during the long flight. Because the flight time was very long and it was not sure if later there could be a gust of wind or some problem, the copter was not controlled to rise too high out of the somewhat wind‐protected area we did the testing in. Since we have remote‐controlled our quadcopters for shorter but still substantial flights where the altitude can be controlled (for example, in the video of a previous prototype https://youtu.be/pns0RaCsMK8), we do not believe our conclusions are significantly altered by this uncertainty.

The longest test was carried out on Sept. 19, 2018. The sun was at the zenith position at latitude 1.5^°^ approximately, while the geographical location was at a latitude of 24.5^°^, so the angle difference is 23^°^. The sun started out at 22.5 ^°^ toward the east at 10:30 am and then 30^°^ toward the west. As a result, the intensity varied approximately from 79% to 92% of the strongest possible, which is about 1100 W m^−2^. On Sept. 19, 2018, the flight location had excellent sunshine (almost no clouds) from 10:00 am to after 2:00 pm. Also, the quadcopter was hovering, maintaining horizontal position for most of the time. Hence, the solar intensity is between 870 and 1000 W m^−2^ approximately. The solar power was more than required near noon, so the battery was recharged to prolong the flight time approaching 2:00 pm when the solar intensity decreased to less than 900 W m^−2^. The resulting power generated from the solar modules then varies in the range between 217 and 250 W (assuming 18% efficiency of the solar panels, as by that time they had suffered through multiple flights and repeated landings), also plotted in Figure 1a, line (g), for easy comparison with the theoretical curves. As shown in line (g), this power range only corresponds to a relatively small variation of the PWR and with *r* > 0.2 always. The power range is above the recorded average of the flight (21 5 W) because the solar panels were not operated at the maximum power point. Also, the orientation of the quadcopter would influence the insolation, but it was much more dynamic. Because a hovering quadcopter does not deviate from the horizontal level for a large angle, we expect it is less of an influence compared to the position of the sun in the sky.

Our model in this paper should be applicable for quadcopters of different mass. What we report here are the smaller ones that can possibly fly for the longest times. A larger solar quadcopter could give more room for solar panels and be lifted with larger motors of higher efficacy, so we expect longer durations possible in even larger quadcopters because even a lower solar intensity could fulfill the requirements. In the past, Kingry et al. developed a comparatively heavy solar quadcopter^[^ ^44^ ^]^ with an exceptionally sturdy frame. Its weight is 8175 g, in contrast to ours of 2230 and 2350 g, respectively, for the two demonstrations reported here. The Kingry aircraft's solar cells required 2200 in^2^ of surface area, similar to our aircraft (2150 in^2^), so if we compare the weight of the two aircraft on this basis, then the Kingry design resulted in 0.27 in^2^ g^−1^ of available surface area, whereas our design resulted in 0.86 in^2^ g^−1^. Our design thus requires substantially less thrust during operation, but is obviously more fragile. The Kingry design made use of an customized flight dynamics program, but we found that our copters could fly well with an existing flight control program (pixhawk). Only proportional, integral, derivative parameters needed to be adjusted, so the development of a solar quadcopter was simplified.

## 3 Conclusion

In conclusion, solar quadcopters were designed based on a concise model which is able to simplify the analysis of the motor power requirements and solar power generation. Quadcopters with on‐board solar panels were discovered to have severe fluctuations of voltage, current, and power, and these are worse for long duration flights. A small battery substantially improves this, contributing little to the actual power flow but smoothing voltage variation to less than 5%. The fluctuations of current and hence power were also significantly reduced. Finally, an 86‐cell quadcopter making use of a small 18 350 6S1P battery (881 mAh) was airborne for 208 min, demonstrating that the use of solar power can extend a drone's endurance by 48.7 times from the battery alone. The total energy generated by the solar module was 33 300 mAh, and had this come from a 6S battery, it would have weighed more than 3 times more than the solar module itself.

## 4 Experimental Section

The 5S and 6S fuselage and solar modules were all made in the lab.

##### Fuselage and Electronics

The same fuselage was used for both quadcopters, and has a frame size of 164 cm (distance between two diagonal motor centers). Four round 3K carbon‐fiber tubes of 10 mm diameter (inner diameter 8 mm) were used to form four arms that connect a pair of center boards and the motor mounts. The center boards (top and bottom ones) were with a diameter of 14 cm, designed in the lab, then cut and drilled with holes from a 1.5 mm thick 3K carbon‐fiber board using a computer numerical control milling machine. The holes were made to hold the carbon‐fiber tubes and electronics, like electronic speed controller (ESC), flight controller, receiver, and so on. The carbon‐fiber tubes were fixed to the center boards and the motor mounts using small c‐clamps. Each tube was 74 cm long. Four tubes, the center boards, and the motor mounts form the main body with a frame size of 164 cm. To make the fuselage firm, an X‐shape formed using carbon‐fiber sticks was attached below the four arms with 3D‐printed joints. The carbon‐fiber sticks are 1 m long with a diameter of 4 mm. The 3D‐printed materials are commercially available polylactide (PLA), which shows sufficient strength for the quadcopters with 164 cm frame size to survive many hours of operation.

Four MAD5005 motors were used. According to the specifications of such motors,^[^ ^42^ ^]^ the 18 in. ones have the best efficacy, compared to smaller propellers, so this size was chosen. In addition, an 18 in. foldable propeller has been tested to have the least power consumption among several 18 in. propellers. The propeller specification is 17.5 in.,^[^ ^45^ ^]^ but the diameter is actually 18 in. with the propeller mount included. A 4 in. 1 ESC was used to control the motor speed. The flight controller was Pixhawk with the Ardupilot program. The telemetry data link was also set to transmit flight parameters to the ground station (a smart phone) for monitoring and recording. The flight controller, the RF receiver, the GPS module, and the telemetry data link were all operating at 5 V, so a power module was used to convert the solar module voltage to 5 V.

##### 5S and 6S Solar Modules

SunPower Maxeon 5 in. c‐Si solar cells were used. Each solar module was divided into five sections. The center one was an array of 6 × 6 solar cells. The other four sections were placed to four sides and made foldable, so the entire solar quadcopter could be easily placed in a hatchback for transport to a location with good sunshine, prescheduled according to the weather forecast.

For the 5S quadcopter, the four side sections all have 5 × 2 solar cells. Including the center piece, there are 76 solar cells, split electrically into two parallel modules. Each has 38 Si solar cells connected in series to give a voltage between 18.5 and 21 V, corresponding to a 5S specification. The layout is shown in Figure 2b.

For the 6S quadcopter, two of the side sections have 5 × 2 solar cells and the other two have 5 × 3 solar cells. The two with 5 × 2 solar cells are placed at the front and back sides and the other two are placed on the left and the right sides. Thus the total number of solar cells is 86. Again, they are divided electrically into two parallel modules. Each has 43 solar cells connected in series for to give a voltage between 22.2 and 25.2 V, corresponding to the 6S specification. The layout is shown in Figure 2c.

In the above two parallel modules, a blocking diode is connected in series with each so as to prevent the current from flowing back to the solar cells.

##### Supporting Frames for Solar Modules

**Figure 5:**
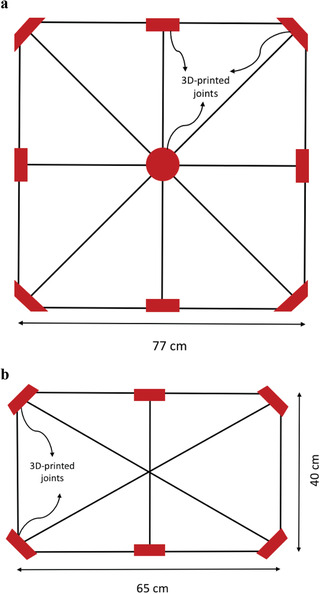
Supporting frames: a) the center frame of 6 × 6 solar cells with a shape of a) British Union Jack; b) the frame design of side modules of 5 × 3 solar cells. (The 3D‐printed joints shown in this figure are not to scale for convenience of illustration.)

All the supporting frames for solar modules are made of carbon‐fiber sticks that are connected with 3D‐printed joints. The center one of 6 × 6 solar cells is supported with a shape of British Union Jack within a square of 77 × 77 cm^2^. The arrangement of the sticks is shown in **Figure** **5**a. The side module of 5 × 3 solar cells has a nearly rectangular shape and an asterisk structure, with a size of 65 × 40 cm^2^, as shown in Figure 5b. The side module of 5 × 2 solar modules is similar except with a size of 65 × 27 cm^2^. The frame supporting the center section of solar cells is attached to the quadcopter, also with 3D‐printed joints. The four side sections are attached to frames hinged at side facing the center section such that they can be folded and flipped to the center area to reduce the overall size during transport. Again, the 3D‐printed materials are commercially available PLA and showed sufficient strength to support the modules with 86 5 in. solar cells for the flight of hours. Those fiber‐stick frames and joints increase the weight by 55–60%, compared to the bare SunPower 5 in. c‐Si solar cells, leading to a PWR of 0.268 W g^−1^, which is calculated based on the specification of the solar cells.

The analysis of the theoretical model and the following implementation show that those materials (carbon‐fiber components, 3D‐printed PLA joints, c‐Si solar cells, etc.) enable the fully solar‐powered quadcopters of 164 cm frame size to have light weight and strength for continuous airtime of hours.

##### Statistics

Telemetry from the aircraft, as shown in Figures 3 and 4, is raw data and the curves shown are not fitted ones.  For Figures 3 and 4, the data are read from the screen‐capture videos, which have the information (current, voltage, flight time, etc.) transmitted from the copter to the smart phone. For Figure 4a,b, data points were read at a 30 s interval and connected directly on the plot.  Even with 30 s interval, there are near 300 and over 400 data points for Figure 4a,b, respectively. For Figure 3a, data points were read at a 5 s interval. There are over 200 data points. As the data points are so close together, they are not shown on the plots. For Figure 3b, they are taken from Figure 4a for the first 20 min because Figure 4a is the situation with a battery to stabilize the voltage. As shown in Figure 3a,b, the voltage variation becomes very little.

## Conflict of Interest

The authors declare no conflict of interest.

## Author Contributions

C.‐F.L. and T.‐J.L. carried out most of the fabrication steps of the solar quadcopters and solar modules reported here. C.‐H.C. assisted some fabrication of the solar modules. W.‐S.L. and H.L. carried out the fabrication of other solar quadcopters and performed corresponding flight tests to provide in‐depth information for this study. J.‐Y. Lin investigated the parameters of flight control and electronics for better stabilization. C.‐F. Lin derived the concise model, performed the flight tests and data analysis for this report and wrote the manuscript. T.‐J.L. also helped part of the data analysis. A.D. provided the data for the Singapore quadcopters and helped improve the manuscript.

## Supporting information

Supporting Information

## References

[1] C. S. Paul , B. J. Kathryn , A. B. Marilyn , S. Linda , L. V. Edward , L. Loren , Nat. Energy 2016, 1, 16043.

[2] J. H. Lehr , J. Keeley , Alternative Energy and Shale Gas Encyclopedia, John Wiley & Sons, 2016.

[3] US Energy Information Administration , International Energy Outlook 2016, CreateSpace Independent Publishing Platform, 2016.

[4] R. J. Boucher , J. Aircr. 1985, 22, 840.

[5] B. E. Layton , Int. J. Green Energy 2008, 5, 438.

[6] V. K. Kostiantyn , P. Bhauriyal , L. Piveteau , C. P. Guntlin , B. Pathak , M. V. Kovalenko , Nat. Commun. 2018, 9, 4469. 30367050 10.1038/s41467-018-06923-6 PMC6203722

[7] D. B. Sandalow , Plug-In Electric Vehicles: What Role for Washington?, Brookings Institution Press, 2019.

[8] Sunpower , Maxeon Gen III Solar Cells. Datasheet, 2020, https://us.sunpower.com/sites/default/files/media-library/spec-sheets/sp-sunpower-maxeon-solar-cells-gen3.pdf. (accessed: June 2020).

[9] N. T. Jafferis , E. F. Helbling , M. Karpelson , R. J. Wood , Nature 2019, 570, 491. 31243384 10.1038/s41586-019-1322-0

[10] C. S. Goh , J. R. Kuan , J. H. Yeo , B. S. Teo , A. Danner , Prog. Photovolt. Res. Appl. 2019, 27, 869.

[11] C. S. Goh , J. R. Kuan , J. H. Yeo , B. S. Teo , A. Danner , IEEE 46th Photovoltaic Specialists Conference (PVSC), IEEE, Piscataway, NJ 2019, p. 2829.

[12] S. Jordan , T. DeGraw , M. Mahon , G. Murphy , I. Chopra , V. T. Nagaraj , 73rd Annual AHS International Forum and Technology Display 2017 (AHS Forum 73), Texas May 2017.

[13] X. Z. Gao , Z. X. Hou , Z. Guo , J. X. Liu , X. Q. Chen , Energy Convers. Manage. 2013, 70, 20.

[14] X. F. Zhu , Z. Guo , Z. X. Houn , Prog. Aerosp. Sci. 2014, 71, 36.

[15] G. Abbe , H. Smith , Renewable Sustainable Energy Rev. 2016, 60, 770.

[16] S. Li , W. Zhou , X. Wang , Mater. Sci. Eng. 2017, 187, 012011.

[17] M. Hassanalian , D. Rice , A. Abdelkefi , 2018 AIAA Aerospace Sciences Meeting AIAA, Reston VA 2018, p. 1.

[18] W. H. Maya , Inside the First Solar‐Powered Flight Around the World, Smithsonian Magazine, 2018.

[19] M. Hassanalian , A. Abdelkefi , Prog. Aerosp. Sci. 2017, 91, 99.

[20] L. Petricca , P. Ohlckers , C. Grinde , Int. J. Aerosp. Eng. 2011, 2011, 1.

[21] R. Hallion , Taking Flight: Inventing the Aerial Age, from Antiquity Through the First World War, Oxford University Press, Oxford, England 2003.

[22] Greg , Goebel, https://web.archive.org/web/20110629140626/http:/www.vectorsite.net/avheli_1.html (accessed: March 2020).

[23] M. Saska , J. Vakula , L. Preucil , IEEE ICRA, IEEE, Piscataway, NJ 2014, p. 3570.

[24] T. S. No , Y. D. Kim , M. J. Tahk , G. E. Jeon , Aerosp. Sci. Technol. 2011, 15, 431.

[25] M. Saska , V. Vonasek , T. Krajnik , L. Preucil , Int. J. Rob. Res. 2014, 33, 1393.

[26] M. L. Prabha , R. Thottungal , S. Kaliappan , Int. J. Adv. Eng. Emerging Technol. 2016, 8, 303.

[27] G. Ostojić , S. Stankovski , B. Tejić , N. Đukić , S. Tegeltija , Int. J. Ind. Eng. Manag. 2015, 6, 43.

[28] J. H. Kim , M. S. Kang , S. D. Park , J. Intell. Rob. Syst. 2010, 57, 9.

[29] E. Kuantama , I. Tarca , S. Dzitac , I. Dzitac , R. Tarca , Symmetry 2018, 10, 291.

[30] I. Penkov , D. Aleksandrov , Int. J. Automot. Mech. Eng. 2017, 14, 4486.

[31] A. Gibiansky , http://andrew.gibiansky.com/downloads/pdf/Quadcopter%20Dynamics,%20Simulation,%20and%20Control.pdf (accessed: February 2020).

[32] M. Khan , Int. J. Sci. Technol. Res. 2014, 3, 130.

[33] J. Doyle , https://solarbay.com.au/size-weight-solar-panels/ (accessed: January 2020).

[34] J. Zhao , A. Wang , M. A. Green , Photovoltaics Res. Appl. 1999, 7, 471.

[35] S. C. Shiu , J. J. Chao , S. C. Hung , C. L. Yeh , C. F. Lin , Chem. Mater. 2010, 22, 3108.

[36] J. F. Geisz , M. A. Steiner , N. Jain , K. L. Schulte , R. M. France , W. E. McMahon , E. E. Perl , D. J. Friedman , IEEE J. Photovoltaics 2018, 8, 626.

[37] M. Steiner , G. Siefer , T. Schmidt , M. Wiesenfarth , F. Dimroth , W. B. Andreas , IEEE J. Photovoltaics 2016, 6, 1020.

[38] M. A. Green , Y. Hishikawa , E. D. Dunlop , D. H. Levi , H. E. Jochen , M. Yoshita , H. B. Anita , Prog. Photovoltaics 2019, 27, 565.

[39] F. Haase , C. Hollemann , S. Schäfer , A. Merkle , M. Rienäcker , J. Krügener , R. Brendel , R. Peibst , Sol. Energy Mater. Sol. Cells 2018, 186, 184.

[40] M. A. Green , Y. Hishikawa , W. Warta , E. D. Dunlop , D. H. Levi , H. E. Jochen , H. B. Anita , Prog. Photovoltaics 2017, 25, 668.

[41] M. Nakamura , K. Yamaguchi , Y. Kimoto , Y. Yasaki , T. Kato , H. Sugimoto , IEEE J. Photovoltaics 2019, 99, 1863.

[42] http://madcomponents.co/index.php/mad5005-280kv (accessed: June 2020).

[43] M. Seyedmahmoudian , B. Horan , T. K. Soon , R. Rahmani , A. M. Than Oo , S. Mekhilef , A. Stojcevski , Renewable Sustainable Energy Rev. 2016, 64, 435.

[44] N. Kingry , L. Towers , Y. C. Liu , Y. Zu , Y. Wang , B. Staheli , Y. Katagiri , S. Cook , R. Dai , IEEE International Conference on Robotics and Automation (ICRA), IEEE, Piscataway, NJ 2018, pp. 1251–258.

[45] http://tarotrc.com/Product/Detail.aspx?Lang=en&Id=d1b23db2‐07f4‐4587‐8abf‐ee711df578b1 (accessed: June 2020).

