# Supplementary material for: Solar Power Can Substantially Prolong Maximum Achievable Airtime of Quadcopter Drones
Source: Adv Sci (Weinh). 2020 Aug 19;7(20):2001497. doi: 10.1002/advs.202001497 (PMC7578872; doi:10.1002/advs.202001497)
Supplement: Supplementary file 1 — Supporting Information [file ADVS-7-2001497-s001.pdf]

© 2020 Wiley-VCH GmbH

ADVANCED SCIENCE
Open Access

Supporting Information

for *Adv. Sci.*, DOI: 10.1002/advs.202001497

Solar Power Can Substantially Prolong Maximum Achievable Airtime of Quadcopter Drones

Ching-Fuh Lin, Ta-Jung Lin, Wei-Sheng Liao, Hsiang Lan, Jiun-Yu Lin, Chi-Han Chiu, and Aaron Danner*

Supporting Information

Evaluation of power consumption and flight time of quadcopter

Derivation of quadcopter power requirements

To clearly evaluate quadcopter power consumption and production from any power source, including solar power, it will be of great help to formulate a relationship between power and aircraft weight from fundamental physical principles, as will be shown. The lifting force in a rotorcraft is provided by downward airflow, with the thrust produced by motor-driven propellers. Such an airflow carries a certain amount of momentum and downward kinetic energy per unit time. Assuming that the area of the air flow is A, the air density is ρ, and the average downward speed of an air molecule is v, within a time duration $\Delta\tau$, then the total mass of the air propelled downwards over this interval is $Av\Delta\tau\rho$. The downward kinetic energy KE that this air mass acquires is given by $KE = (1/2)\ (Av\Delta\tau\rho)v^2$ and the power P which must be provided to acquire this energy is $P = KE/\Delta\tau = (1/2)\ (A\rho)v^3$. Also, the momentum acquired, Δp, when this air mass is accelerated from an average speed of zero to v, is equal to $(Av\Delta\tau\rho)v$, leading to a lifting force F given by

$$F = \Delta p/\Delta\tau = (A\rho)v^2 \quad \text{(S1)}$$

Equation (S1) is the same as Equation (1) in Reference [1], but different from a derived formula, $v = \sqrt{\frac{F}{2A\rho}}$, based on reference [2]. From Equation (S1), a simple relation can be given: P/F = (1/2) v. Also, from the relation of F and v, we are able to obtain the relation between the power P and the lifting force F of the downward air flow: $P = \frac{1}{2}\frac{1}{\sqrt{A\rho}}F^{3/2}$, which is slightly different by a proportional constant from the formula given in reference [2].

In electrical copters, the power is provided by propellers that are further driven by the motors through the input of electrical power. Several routes of losses, including motor stator and rotor core loss,[3] copper loss,[4] propeller efficiency loss,[5] and so on, lead to a conversion efficiency of input power to downward air-flow power (thrust) less than 100%. In addition, the electrical motor power is usually provided by an electronic speed controller (ESC), which does not have a 100% efficiency of converting input electrical power to the waveform needed by the motors,[6] either. In any case, taking into account all losses, we can simply write $P = \eta P_e$, where

P_e is the input electrical power to the system and η is the overall conversion efficiency. Then the relation between the input electrical power P_e and the lifting force F of the air flow is given by

$$P_e = \frac{1}{2\eta}\frac{1}{\sqrt{A\rho}}F^{3/2} \qquad \text{(S2)}$$

This lifting force F is equal to the drone weight, in terms of gravitational force, if the drone is in static hover. If the drone is unbalanced or moving, then more power than that needed to overcome gravity would be needed, so required $P_e \geq \frac{1}{2\eta}\frac{1}{\sqrt{A\rho}}F^{3/2}$.

Confirmation of the relation between the input electrical power and the lifting force with a real motor

According to Equation (S2), the required input electrical power super-linearly increases with the lifting force, which cannot be less than the total weight of the copter. Figure S1 shows a fitted theoretical Equation (S2) curve and a measurement of a commercial MAD5005 motor. The assumed air density is 1.225 kg m^{-3}. For Figure S1, the propeller used was 18 inches in

diameter with an area swept of 0.164 m^2, and the efficiency η was determined to be 47 %. In the measurement, the spin rate of the motor was controlled through the ESC with its power input from a 6S battery. The applied voltage and current, monitored through a voltage meter and a current meter, to the ESC were recorded and the power was calculated accordingly. Because a large-capacity battery was used, the voltage could maintain at 23.0 V during the measurement period of time, which was not long. The lifting force, measured using a weight scale attached to the motor with the propeller, was also recorded simultaneously at a certain voltage and current. The measurement curve and the theoretical curve match well with a constant efficiency, so we did not use a voltage-dependent efficiency. There is only a small discrepancy between the two curves, indicating this simple model can predict power requirements pretty well. The measured power consumption increases at a slightly lower rate than the theoretical prediction. When the power increases, two opposing effects may be happening. One is that motors fast-spinning motors propel air molecules to a higher ultimate velocity, making the air stream more directional and hence increasing the overall efficiency.[7] The other is that extra loss could be induced through an increased motor temperature at high-power consumption levels, decreasing the overall efficiency.[8] According to Figure S1, the second effect plays less of a role than the

first one, probably due to the excellent thermal properties of the motor.

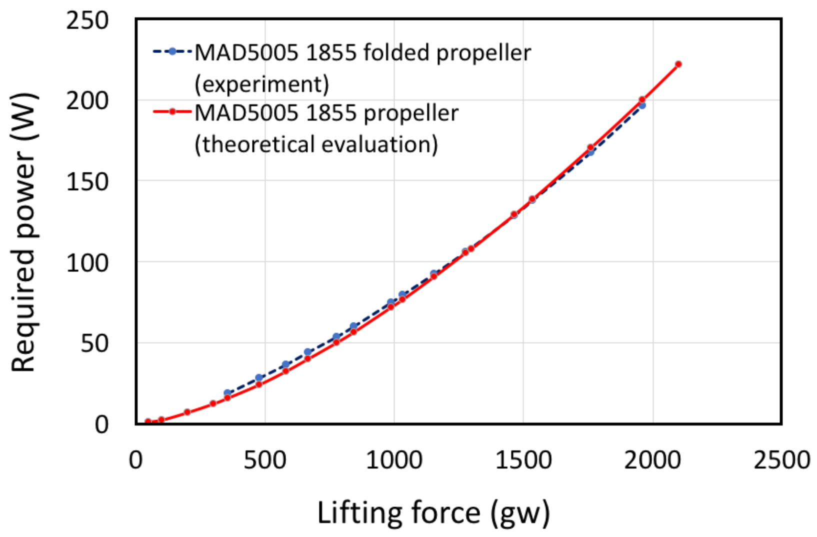

Figure S1. Comparison of required power vs. lifting force between an experimental and theoretical evaluation.

For multi-copters, there is more than one motor. Assuming n motors and n corresponding propellers, the total lifting force is $F_t = nF$ and the total power consumption is $P_t = nP_e$, while the total area swept by the propellers $A_t = nA$, leading to the following equation:

$$P_t = \frac{1}{2\eta}\frac{1}{\sqrt{A_t\rho}}{F_t}^{3/2} \qquad \text{(S3)}$$

Equation (S3) has the same form as Equation (S2), but with more motors, each can

operate at greater efficiency when sharing only 1/n of the total power if sized correctly. This factor is reflected by the area A_t in Equation (S3), which is n times larger than that of the single motor with a single propeller.

With Equation (S3), a battery-powered quadcopter can first be considered. For a battery, the stored energy E is proportional to its weight W_b, $E = kW_b$, with k around 0.2 Wh g^{-1} in a lithium battery. The airtime that the battery can provide is given by E/P_t, where P_t is the required power shown in Equation (S3). The total weight of the copter is the fuselage weight W_f plus the battery weight W_b. The lifting force is no less than the total weight. The maximum airtime T is obtained when the total lifting force F_t is equal to the copter weight, $F_t = W_f + W_b = W_f + E/k$. Thus the maximum flight time T is given by

$$T = 2\eta\sqrt{A_t\rho}\,\frac{E}{(W_f+E/k)^{3/2}} \quad \text{(S4)}$$

Based on Equation (S4), the flight time of a quadcopter using an ordinary battery can be estimated. As shown in Figure S2, the flight time does not always increase with battery mass, because the consumed power super-linearly increases with total copter weight. This is a major

limitation of batteries. However, if the power is provided from sunlight, the flight time can be greatly increased.

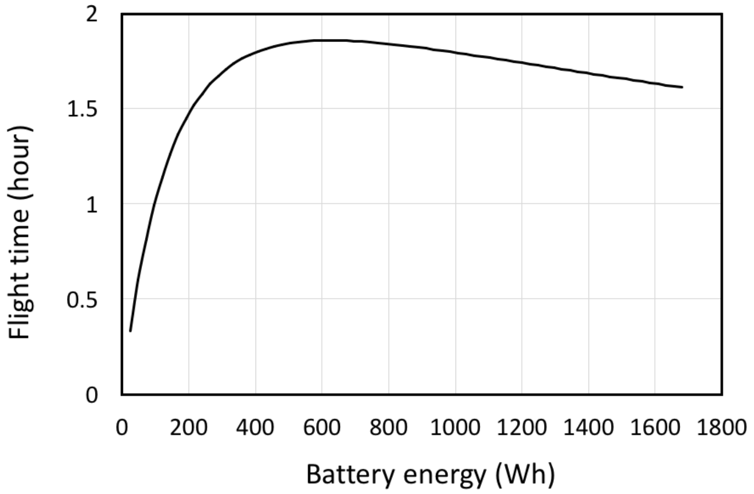

Figure S2. Flight time vs. Battery energy.

In the case of sunlight, the power acquired is proportional to the surface area of the solar modules, while energy delivered is proportional to time. The consideration of power is more meaningful than that of energy for drones. Because the solar cells have a fixed thickness, their weight is proportional to their surface area. This makes the output power (P_s) proportional to the weight of the solar cells (W_s), $P_s = rW_s$, where r is the power-to-weight ratio (PWR), the most critical parameter in determining the possibility of using sunlight as the main power source in rotorcraft. For typical c-Si solar cells with a thickness of 200 μm, r = 0.429 W g^{-1} under the

standard solar intensity of 100 mW cm^{-2} at 20% efficiency. When combining solar cells into modules, r is reduced because packaging and/or a supporting frame add to their weight. The relation between the output power of the solar module and the total aircraft weight (W) including its solar module is then given by

$$P_s = rW_s = r(W-W_f) \qquad (S5)$$

where W_f is the fuselage weight.

Basic specifications of the fabricated solar quadcopters

The 5” SunPower c-Si solar cell has an efficiency of about 20%, so it can generate 3.2 W under the standard solar intensity. The total power could be 243 W and 275 W for a 5S solar module with 76 solar cells or a 6S solar module with 86 solar cells, respectively. The fuselage for both modules weighs 1330g, while the total quadcopter weight is 2235 g and 2354 g in the case of the 5S solar module and 6S solar module, respectively. According to Equation (S2), the required power for the four motors is 122 W and 131 W, respectively. Such power requirements

are almost nearly half what the solar modules can optimally deliver. However, when the solar modules are fabricated by hand, there are scratches, imperfect orientation, imperfect soldering, and other solar cell loss mechanisms. Also, the solar modules might not operate at their maximum power points and their orientation will not be optimal due to the attitude changes of the copters[9] and the position of the sun during flights. Hence the actual power is substantially reduced, but with healthy margin to account for such loss, we expect the quadcopters to be substantially solar powered. The quadcopters were built with a 5S or 6S voltage, so a power module is used to convert this to 5 V for the electronics, including the pixhawk flight controller, the rf receiver, the GPS module, the telemetry data link, etc. The motors are brushless. A 4-in-1 electronic speed controller (ESC) is used to control the four motors.

Estimation of solar quadcopter flight time

To stabilize the operation voltage of a solar quadcopter, a battery is used together with the solar module. Then the power supply can be viewed as a hybrid system. Here let's evaluate the flight time with this hybrid energy source. Assume that the total current required for the copter is Y amperes and the battery has X amp-hours of useful charge, so the battery-only flight time would be $T_b = X/Y$. If the solar module fractionally provides z of the

total current, the current consumed from the battery would be (1-z)Y amperes. Then the battery will last longer, to a total time of $T_h = X/[(1-z)Y]$, extending flight by a factor of $EF = T_h/T_b = 1/(1-z)$. Figure S3 shows the relation between the extended factor (EF) and the ratio of current provided by the solar module. The extended factor becomes significant only when the ratio is above 90%.

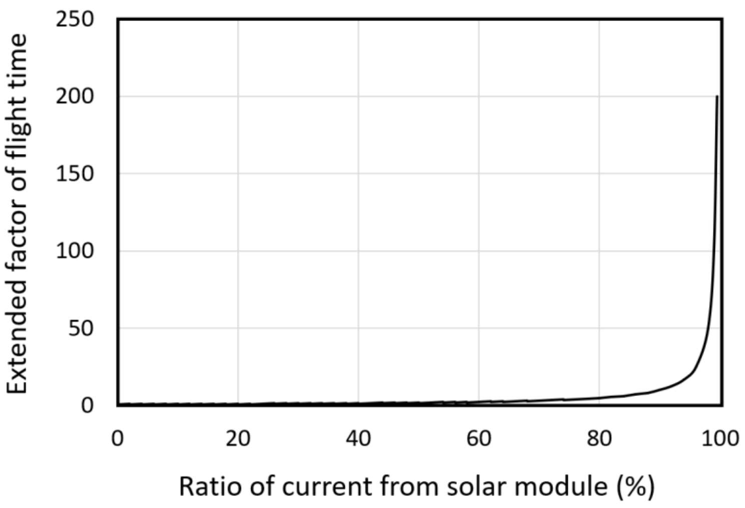

Figure S3. Extended factor (EF) vs. ratio of current provided by the solar module.

References

1. Khan, M. (2014). Quadcopter Flight Dynamics. *International Journal of Scientific & Technology Research* **3**, 130–135.

2. Gibiansky, A. Quadcopter Dynamics, Simulation, and Control.

http://andrew.gibiansky.com/downloads/pdf/Quadcopter%20Dynamics,%20Simulation,%20and%20Control.pdf

3. Sato, T., Enokizono, M. (2018). Evaluation of stator core loss of high speed motor by using thermography camera. *AIP Advances* **8**, 047609.
4. Katagiri, H., Semba, K., Sano, H., Yamada, T. *Fast calculation of copper loss in three-phase synchronous motor by zooming method* (ISEF, 2017)
5. Gur, O. (2014). Maximum Propeller Efficiency Estimation. *Journal of Aircraft* **51**,2035–2038.
6. Gong, A., Verstraete, D. *Experimental Testing of Electronic Speed Controllers for UAVs* (53rd AIAA/SAE/ASEE Joint Propulsion Conference, 2017).
7. Penkov, I. and Aleksandrov, D. (2017). Propeller shrouding influence on lift force of mini unmanned quadcopter. *International Journal of Automotive and Mechanical Engineering* **14**, 4486–4495.
8. Yoon, M. K., Jeon, C. S. and Kauh, S. K. (2002). Efficiency Increase of an Induction Motor by Improving Cooling Performance . *IEEE Transactions on Energy Conversion* **17**, 1–6.
9. Dörenkämper, M., Der Werf, D. V., Sinapis, K., De Jong, M. M. and Folkerts, W.

Influence of wave induced movement on the performance of floating PV systems. In *2019 36th European Photovoltaic Solar Energy Conference and Exhibition*, Paper 6DO.9.1 (WIP Renewable Energies, 2019).
